# Phase-Resolved Optical Coherence Elastography: An Insight into Tissue Displacement Estimation

**DOI:** 10.3390/s23083974

**Published:** 2023-04-13

**Authors:** Ana Batista, Pedro Serranho, Mário J. Santos, Carlos Correia, José P. Domingues, Custódio Loureiro, João Cardoso, Sílvia Barbeiro, Miguel Morgado, Rui Bernardes

**Affiliations:** 1 Coimbra Institute for Biomedical Imaging and Translational Research (CIBIT), Institute for Nuclear Sciences Applied to Health (ICNAS), University of Coimbra, 3000-548 Coimbra, Portugal; 2 Department of Physics, Faculty of Science and Technology, University of Coimbra, 3004-531 Coimbra, Portugal; 3 Department of Science and Technology, Mathematics Section, Aberta University, 1250-100 Lisbon, Portugal; 4 Department of Electrical and Computer Engineering, Faculty of Science and Technology, University of Coimbra, 3004-531 Coimbra, Portugal; 5 Department of Mathematics, CMUC, University of Coimbra, 3004-531 Coimbra, Portugal; 6 Faculty of Medicine (FMUC), Clinical Academic Center of Coimbra (CACC), University of Coimbra, 3000-143 Coimbra, Portugal

**Keywords:** optical coherence elastography, phase-resolved OCE, displacement estimation, Young’s modulus

## Abstract

Robust methods to compute tissue displacements in optical coherence elastography (OCE) data are paramount, as they play a significant role in the accuracy of tissue elastic properties estimation. In this study, the accuracy of different phase estimators was evaluated on simulated OCE data, where the displacements can be accurately set, and on real data. Displacement (∆d) estimates were computed from (i) the original interferogram data ${(\Delta \varphi }^{ori})$ and two phase-invariant mathematical manipulations of the interferogram: (ii) its first-order derivative (${\Delta \varphi }^{d}$) and (iii) its integral (${\Delta \varphi }^{int}$). We observed a dependence of the phase difference estimation accuracy on the initial depth location of the scatterer and the magnitude of the tissue displacement. However, by combining the three phase-difference estimates (${\Delta d}^{av})$, the error in phase difference estimation could be minimized. By using ${\Delta d}^{av}$, the median root-mean-square error associated with displacement prediction in simulated OCE data was reduced by 85% and 70% in data with and without noise, respectively, in relation to the traditional estimate. Furthermore, a modest improvement in the minimum detectable displacement in real OCE data was also observed, particularly in data with low signal-to-noise ratios. The feasibility of using ${\Delta d}^{av}$ to estimate agarose phantoms’ Young’s modulus is illustrated.

## 1. Introduction

Evaluation of tissue biomechanical properties can provide important information for disease diagnosis and monitoring. Tissue stiffness has long been recognized as a biomarker of disease. Its assessment by palpation was already performed in ancient Egypt, and it is still a common tool for physical diagnosis in clinical practice [1]. Quantitative assessment of tissue biomechanics can be performed non-invasively using elastography techniques, including optical coherence elastography (OCE).

First introduced in 1998 by J. M. Schmitt [2], OCE is still an emerging technique for assessing tissue elasticity. This imaging modality combines optical coherence tomography (OCT) with a locally applied force to induce tissue displacement [3]. There are different implementations of OCE, varying the type of mechanical loading of the tissue (e.g., contact or non-contact, static, quasi-static, or dynamic, localized or global), the OCT scanning protocol, and the method used to quantify tissue deformation [3,4,5,6]. Nonetheless, they are all based on the detection of tissue deformation from consecutive OCT scans. 

Speckle-tracking or phase-resolved techniques can be used to obtain tissue displacements. Speckle-tracking methods are based on intensity information and, therefore, typically require large displacements, making them unsuitable for imaging fragile tissues [7]. Phase-resolved measurements instead use phase difference information [6,7]. This provides nanoscale displacement sensitivity [3]. Combined with the microscale spatial resolution of OCT systems, OCE enables measurement scales unattainable by other elastography methods [3]. Therefore, its application in medical research has gained momentum, especially in ophthalmology [8,9,10].

The system’s phase stability is a determinant for the measurement precision of phase-resolved OCE. Imperfect timing synchronization is the primary source of phase instability [11]. This problem is more relevant for swept-source systems due to the frequency jitter introduced by wavelength sweeping with mechanically moving mirrors. Several approaches have been considered and implemented to minimize the timing errors that lead to phase jumps. Commonly used solutions involve the generation of optical timing references. To correct for the nonlinearity of the swept-source frequency sweeps, Mach-Zehnder Interferometer (MZI) optical clocks are typically used. Fiber Bragg Gratings (FBG) are used as a wavelength-dependent trigger signal to synchronize the source sweep with the data digitization by the data acquisition (DAQ) board [11]. Other solutions include the implementation of a second reference arm to measure time-induced phase variations, as proposed by Vacok et al. [12]. The authors greatly improved the system’s sensitivity by calibrating the phase using the recorded phase noise [12]. Recently, Li et al. described a 40-fold increase in phase stability by using a common-path OCE configuration, achieving displacement sensitivities of 0.3 nm [13]. These configurations significantly improve the phase stability of the system and, consequently, the precision of phase-resolved measurements. However, they also require changes to the instrument’s setup that are not always possible or desirable.

The precision and accuracy of the method used to estimate the phase difference also play an important role in the correct assessment of tissue elasticity [6]. Several methods have been proposed to obtain the phase difference from successive interferograms. The most direct approach is obtained by subtracting the phases of consecutive interferograms acquired at the same location within a short period of time [6,7], but this is very sensitive to noise [7]. To increase the signal-to-noise ratio (SNR) and stability, a 2D autocorrelation approach has been proposed by Loupas et al. [14]. This method exploits depth-wise phase information for phase difference estimation. The size of the axial window is variable. However, to obtain an adequate SNR improvement while maintaining a high displacement axial resolution, it cannot be greater than 2% of the imaging depth [7]. Simultaneous averaging of the phase difference in the lateral and axial directions using a rectangular window has also been proposed to increase the robustness of the estimation [15]. This approach has been combined with intensity-based pixel-scale displacement tracking (speckle-tracking) to reduce additive noise and displacement-related decorrelation [15]. Phase averaging combined with a vector method has also been proposed, where incremental vectors of lateral and vertical phase variations are generated from the complex-valued signals to further reduce additive noise and eliminate the need for phase unwrapping [16,17].

The long-term goal of our research efforts is the early detection of neurodegeneration, namely the detection of Alzheimer’s disease (AD) in its asymptomatic phase, by measuring changes in the mechanical properties of the retina, the visible part of the central nervous system. Significant changes in the brain’s elastic properties have already been detected in AD patients [18,19,20]. Moreover, cell mechanic properties appear to be affected by the accumulation of amyloid beta that is characteristic of AD senile plaques [21]. Our goal is to detect subtle changes in retinal elasticity, highlighting the importance of accurately estimating tissue displacements. The goal is to detect subtle changes in tissue elasticity, highlighting the importance of accurately estimating tissue displacements. Reductions in axial and temporal resolution due to the averaging of signals in depth and lateral directions must also be avoided. 

In this study, we evaluated the performance of the original phase difference estimation method to better understand the influence of the magnitude of tissue displacement as well as the scatterer’s depth location on the accuracy of the estimated phase difference from successive scans and to develop alternative solutions to improve the accuracy of phase difference estimation. In addition to the estimation of the phase difference from the raw interferogram data, we also evaluated the estimates from two phase-invariant mathematical manipulations of the interferogram: (i) the first-order derivative of the original interferogram and (ii) the integral of the original interferogram, and from a combination of the three approaches. The performance of each method was evaluated on numerical simulations of OCE data, with and without noise, and real data. 

## 2. Theory

In phase-resolved measurements, the sample displacement Δd is obtained by estimating the phase difference Δφ [6] as:

(1) $$\Delta d=\Delta \varphi \frac{\lambda_{0}}{4\pi n}$$

where, $\lambda_{0}$ is the center wavelength of the laser and n is the refractive index of the sample. As mentioned above, there are several methods to estimate Δφ[z] at depth z, with different accuracies and sensitivities to noise. 

The A-scan intensity and phase are obtained from the measured interferogram F[k] by the inverse discrete Fourier transform (IDFT). F[k] is a discrete signal with N samples corresponding to the wavenumbers swept by the laser source from $k_{min}$ to $k_{max}$. Hence,

(2) $$f[z]=\sum_{k=0}^{N-1}F[k]e^{j2\pi kz/N}$$

where j is the complex unit and the complex function f is discrete in space z. OCT A-scans are defined by the magnitude of f[z], that is, $A_{scan}\left[z\right]=\left|f\left[z\right]\right|,$ with z=0,…,N−1, and the phase φ[z] given by the angle ∠ of f[z].

Now, considering two successive interferograms, $F_{0}$ and $F_{1}$, acquired at the same lateral location of two instants in time, $t_{0}$ and $t_{1}$, with $t_{1}=t_{0}+∆t$, Δφ[z] can be estimated as [6,7]:

(3) $$\Delta \varphi [z]=\varphi_{1}[z]-\varphi_{0}[z]$$

(4) $$\Delta \varphi \left[z\right]={tan}^{-1}\frac{Im\left\{f_{1}\right\}Re\left\{f_{0}\right\}-Re\left\{f_{1}\right\}Im\left\{f_{0}\right\}}{Re\left\{f_{1}\right\}Re\left\{f_{0}\right\}+Im\left\{f_{1}\right\}Im\left\{f_{0}\right\}}$$

where Im and Re are the imaginary and real parts of the complex function f, respectively. In this formulation, the phase difference is computed using only one evaluation of the inverse tangent function, which improves the numerical accuracy over subtracting the separately computed phases. However, it is still sensitive to noise [7].

In this work, we introduce the estimation of Δφ[z] from phase-difference-invariant mathematical variations of the original interferogram: (i) the first-order derivative and (ii) the integral of the original interferogram, respectively computed using a first-order finite difference approximation of the derivative $F^{d}\left[k\right]\approx F^{\prime }\left[k\right]=(F\left[k+1\right]-F[k-1])/2$ and $F^{int}\left[k\right]=\int_{0}^{k}F(u)du$ approximated by the trapezoidal rule. These approximations were chosen because both are second-order numerical schemes and therefore have similar performance. The phase differences ${\Delta \varphi }^{d}\left[z\right]$ and ${\Delta \varphi }^{int}[z]$ were then calculated from $f^{d}\left[z\right]$ and $f^{int}\left[z\right]$, respectively, using Equation (4) after the IDFT.

## 3. Materials and Methods

### 3.1. System Setup

**Figure 1:**
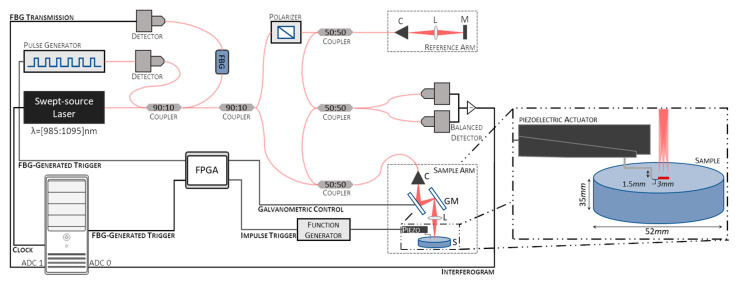
Schematic representation of the system used for the swept-source optical coherence elastography (SS-OCE) system employed. C—Collimator, L—Lens, M—Mirror, GM—Galvanometric mirrors, ADC—Analog-to-digital converter channel.

This study used a home-built swept-source OCE (SS-OCE) system. A schematic representation of the system is shown in Figure 1. It consists of an SS-OCT with a swept-source laser (Axsun, Excelitas Technologies Corp., Mississauga, ON, Canada) emitting at a center wavelength of 1040 nm with a bandwidth of 110 nm and a repetition rate of 100 kHz, coupled with a piezoelectric actuator to induce tissue displacement. The output of the laser source is split by a 90 to 10 optical fiber coupler, with 90% of the light being used to generate the interferograms and the remaining 10% being directed into a FBG. The light reflected from the FBG, at a wavelength of 990 ± 1 nm, is converted to an electrical signal by a photodiode and directed to a digital delay and pulse generator (DG535, Stanford Research Systems, Sunnyvale, CA, USA) to generate trigger pulses with appropriate timing and amplitude for data acquisition. 

The light is further split 90 to 10 between the sample and reference arms to generate the interferograms. In the sample arm, the light is delivered to and collected from the sample using a 50 to 50 optical fiber coupler. An LSM03-BB objective (Thorlabs, Inc., Newton, NJ, USA), with an effective focal length of 36 mm and an entrance pupil diameter of 4 mm, was used to focus the light onto the sample. In the reference arm, the light is reflected from a fixed reference mirror. A fiber polarization controller removes polarization variations between the sample and reference signals. The resulting interference fringes, formed by coupling the sample and reference reflected light using a 50 to 50 optical fiber coupler, are detected by a balanced photodetector that subtracts the two signals to remove the common-mode noise. A DAQ board (X5-400M, Innovative Integration, Inc., Indianapolis, IN, USA) digitizes the photodetector output using the FBG-generated trigger. In addition, the inverted signal of the FBG transmission is recorded in the second analog-to-digital converter (ADC) channel of the DAQ board for further post-processing jitter correction. The clock signal is provided by the internal MZI clock of the laser source. 

Displacements were induced using a piezoelectric actuator (P-287, Physik Instrumente GmbH & Co. KG, Karlsruhe, Germany). The tip of the contact rod is circular, with a diameter of approximately 1.3 mm. It was positioned approximately 1.5 mm to 4.5 mm from the scan line of the OCT beam (Figure 1, inset). Mechanical vibrations were triggered by a transient pulse signal synchronized with the galvanometric mirrors and the FBG-generated trigger to initiate data acquisition using a field-programmable gate array (FPGA). The width, delay, and amplitude of the transient pulse signals were controlled by an arbitrary function generator (AFG3101C, Tektronix, Beaverton, OR, USA).

#### Numerical Simulations

The performance of the phase difference estimators was first tested on numerical simulations of the OCE data. Based on the specifications of the swept-source laser used in our system, simulated interferograms for a single scatterer in a medium with a refractive index of 1.38 were generated using Matlab 2021a (MathWorks^®^, Natick, MA, USA). Considering the spectral intensity distribution of the swept-source laser S(k) and that the refractive index n was constant with depth, the simulated interference signals I(k) were generated as [22]:

(5) $$I\left(k\right)=S(k){\left[a_{R}e^{ik2r}+\int_{0}^{\infty }a(z)e^{ik2(r+nz)}dz\right]}^{2}$$

where k is the wavenumber swept by the laser, ranging between $k_{min}$ and $k_{max}$ within N discrete samples, $a_{R}$ stands for the amplitude of the reference, a(z) is the backscatter amplitude of the object at depth z and, 2r and 2(r+z) are the path lengths in the reference and object arms, respectively. The autocorrelation term resulting from Equation (5) was neglected. The IDFT was then used to retrieve the intensity and phase of the simulated interferogram. The scatter’s depth location is precisely adjusted by changing the length of the object’s arm 2(r+z). A depth-wise shift of 90 nm in each direction was imposed to estimate the phase difference between the two interfaces. In these simulations, the interface shift was limited to ±90 nm relative to the initial location to avoid consideration of phase unwrapping. 

**Figure 2:**
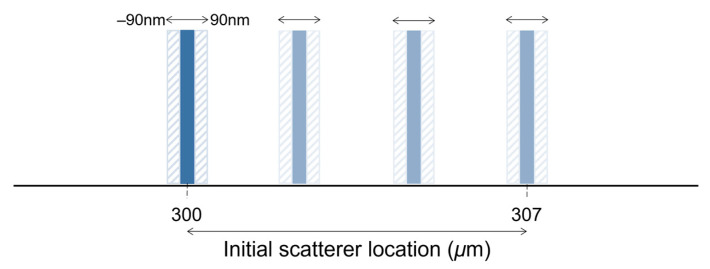
Simplified representation of the displacement simulation.

The theoretical axial resolution of a swept-source system with the above specifications in a medium with a refractive index of 1.38 is approximately 6.7 μm. To better simulate the results obtained with this system, the initial location of the scatterer was moved 7 μm in steps of 10 nm, comprising 710 depth locations (Figure 2). A depth range between 300 μm and 307 μm was chosen because it corresponds to the highest SNR of the system described above. For each location, the scatterer was shifted over a range of 180 nm, and the phase difference was computed. The error in phase difference estimation of each method was evaluated under ideal conditions (no noise) and after adding the system noise (real data) to the generated interferogram. The system noise was approximately 47.73 LSBrms (least significant bit root-mean-square). The original and the shifted interferograms were subjected to random noise of the same level.

### 3.2. Phase Difference Measurements

**Figure 3:**
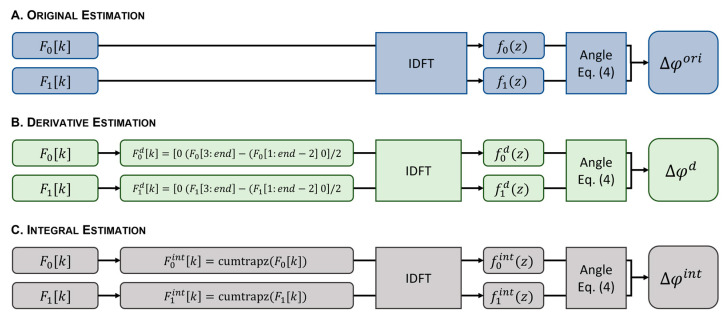
Workflow diagram showing the phase difference estimation from the original interferogram (**A**), the first-order derivative (**B**), and the integral of the original interferogram (**C**). Equation (4): $\Delta \varphi \left[z\right]={tan}^{-1}\left(\left(Im\left\{f_{1}\right\}Re\left\{f_{0}\right\}-Re\left\{f_{1}\right\}Im\left\{f_{0}\right\}\right)/\left(Re\left\{f_{1}\right\}Re\left\{f_{0}\right\}+Im\left\{f_{1}\right\}Im\left\{f_{0}\right\}\right)\right)$.

All methods were implemented using Matlab 2021a (MathWorks^®^, Natick, MA, USA). The workflow is shown in Figure 3. The original phase difference estimate (${\Delta \varphi }^{ori}$) was obtained after the IDFT of each interferogram to obtain the respective A-scan magnitude and phase. The phase difference between consecutive A-scans was then calculated using Equation (4) (Figure 3A). For the proposed phase difference estimators, the first-order derivative ($F^{d}\left[k\right]$) and integral ($F^{int}\left[k\right]$) of the original interferogram were computed prior to the IDFT. An approximation of $F^{d}\left[k\right]$ was obtained using the difference between the discrete channels (Figure 3B). For an approximation of $F^{int}\left[k\right]$, the cumulative integral using the trapezoidal method was used (Figure 3C). Following the IDFT, ${\Delta \varphi }^{d}$ and ${\Delta \varphi }^{int}$ were estimated from Equation (4). Phase unwrapping was applied to the estimated phase differences ${\Delta \varphi }^{ori}$, ${\Delta \varphi }^{d}$, and ${\Delta \varphi }^{int}$, affecting the displacement estimation error of all methods equally. Equation (1) was used to calculate the displacements ${\Delta d}^{ori}$, ${\Delta d}^{d}$, and ${\Delta d}^{int}$, from the estimated phase differences ${\Delta \varphi }^{ori}$, ${\Delta \varphi }^{d}$, and ${\Delta \varphi }^{int}$, respectively.

### 3.3. Real Data Acquisition

#### 3.3.1. Static Conditions

The OCT interference signal of a highly reflective sample (gold-coated mirror) was used to determine the temporal stability of the system and the performance of each estimator under static (real) conditions, i.e., without inducing any displacement. The influence of the imaging depth, and consequently the SNR, was evaluated by changing the relative depth location of the sample. Data were recorded at optical path differences (OPDs) between 0 μm and 3000 μm in steps of 500 μm, with OPD = 0 μm being the depth location with the highest measured SNR (71.19 dB) and OPD = 3000 μm the lowest (40.16 dB).

The minimum detectable displacements were calculated to determine the resolution of each estimator. Fundamental limitations of the minimum detectable displacement in phase-sensitive measurements are phase stability and the precision of the Δφ computation. The former is limited by the system’s temporal stability and is inherently related to the instrument’s setup. The measured temporal stability of the system was 396.9 ± 46.7 ps [23]. No changes were observed in the system’s temporal stability with a decrease in the system SNR.

The displacement resolution is linearly dependent on the standard deviation of the measured phase difference ($\sigma_{\Delta \varphi }$), as shown in Equation (1) [6]. Thus, the $\sigma_{\Delta \varphi }$ was calculated as a metric of the minimum detectable displacement. The lower the error associated with the displacement estimation, the higher the accuracy. The root-mean-square error (RMSE) was computed to provide additional information about the accuracy of the displacement estimation.

#### 3.3.2. Dynamic Conditions

Data were collected using the Motion-Brightness (M-B) scanning protocol [3]. Axial scans were repeated 768 times at a fixed spatial location to track surface motion at high frame rates, corresponding to a scan time of 7.68 ms per location. B-scans were generated by temporally aligning the different spatial locations. Imaging was performed at 256 lateral locations. Data were acquired over an approximately 3 mm line in steps of 11.72 μm (Figure 1, inset). 

Transient pulses were used to induce motion in homogeneous agarose phantoms. The surface motion was generated once at each spatial location, with a delay of 100 μs (corresponding to 10 interferograms), using transient pulses with widths between 100 μs and 600 μs, in steps of 100 μs, with a fixed amplitude of 50 mV. A total of three measurements were made for each pulse width. The average laser power at the sample arm was approximately 1.06 mW.

The estimator with the highest accuracy in both simulated and static data was then used to obtain the phase difference at the phantom boundary. The surface displacement was calculated using Equation (1) to derive the elastic modulus (Young’s modulus) of the phantom, $E=3\rho C_{s}^{2}$, where ρ is the material density and $C_{s}$ is the shear wave velocity. The refractive index of agarose phantoms was calculated from their concentration c as $n_{p}=0.0014c+1.333$ [24]. 

The propagation velocity of the generated surface waves (Rayleigh waves) $C_{R}$ was obtained from the propagation distance of a transient pulse ∆D during the time ∆t as $C_{R}=∆D/∆t$. The $C_{s}$ was then obtained using the correlation $C_{s}=1.05C_{R}$ [7]. Average cross-correlations between tissue displacement curves over time (*n* = 10) in 0.5 mm increments were used to obtain ∆D. 

### 3.4. Phantom Preparation

Homogeneous agarose phantoms, with tissue-mimicking properties, were prepared by gently mixing agar with distilled water at a high temperature (85 °C) in concentrations of 10 g/L, 15 g/L, and 25 g/L while stirring. Glass microspheres, with diameters of 152 ± 32 μm, were added to the mixture at a concentration of 10 g/L to increase optical scattering. The mixture was then placed in containers for molding and cured for approximately 24 h. The resulting phantoms were cylindrical, with diameters and heights of approximately 5.2 cm and 3.5 cm, respectively.

### 3.5. Statistical Analysis

Statistical differences between the squared errors of the Δφ estimators were assessed using the nonparametric Wilcoxon test for paired samples after the Kolmogorov-Smirnov test was used to reject the normality hypothesis. Differences were considered significant at a significance level of 0.05. 

## 4. Results

### 4.1. Simulation Results

A simulation of the interferogram expected from our OCE system was generated to better calculate the error associated with phase difference estimation using different methods. The initial location of the scatterer varied over 7 μm (710 depth locations).

**Figure 4:**
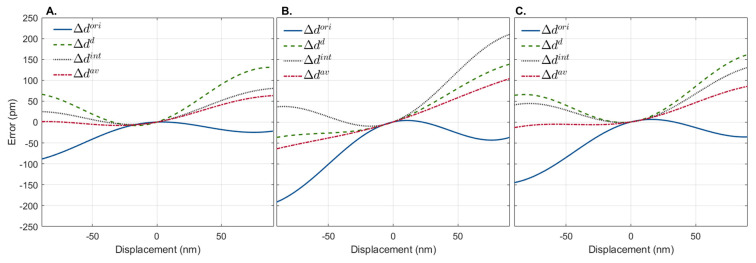
Representative displacement estimation error of simulated interferograms shifted 90 nm depth-wise in both directions using the original interferogram, ${\Delta d}^{ori}$, the first-order derivative, ${\Delta d}^{d}$, and the integral, ${\Delta d}^{int}$ of the generated interferograms. Three depth locations are shown where ${\Delta d}^{ori}$ (**A**), ${\Delta d}^{d}$ (**B**), and ${\Delta d}^{int}$ (**C**) have lower displacement errors. In all cases, the best displacement estimation is achieved by the average of the three methods (${\Delta d}^{av}$).

Each method’s performance depended on the scatterer’s initial depth location. The error, measured as the difference between simulated and measured displacements associated with ${\Delta d}^{ori}$, ${\Delta d}^{d}$, and ${\Delta d}^{int}$, was lower for a total of 267, 204, and 239 depth locations, respectively, out of the 710 depth locations tested. This shows that, depending on the initial depth location of the scatterer, ${\Delta d}^{ori}$ will be a better approach 37.61% of the time, while ${\Delta d}^{d}$ will be a better approach 28.78% of the time, and ${\Delta d}^{int}$ will be a better approach the remaining 33.66% of the time. Figure 4 shows examples of the error associated with displacement estimation at three different depths, where either ${\Delta d}^{ori}$ (Figure 4A), ${\Delta d}^{d}$ (Figure 4B), or ${\Delta d}^{int}$ (Figure 4C) estimators yielded values closer to the real ones (lower error). 

As expected, when zero displacements (static) were simulated, the error associated with the displacement calculation was zero for all methods. Increasing the magnitude of the simulated displacement in any direction decreased the accuracy of all methods. Interestingly, although the error associated with displacement computation increased with distance for all methods, they showed different trends. As shown in Figure 4, the estimation errors for ${\Delta d}^{ori}$ and ${\Delta d}^{d}$ and ${\Delta d}^{int}$ had opposite and complementary trends. As the magnitude of the displacement induced increased, ${\Delta d}^{ori}$ tended to underestimate the displacement, while ${\Delta d}^{d}$ and ${\Delta d}^{int}$ tended to overestimate it. In view of this, we propose a novel displacement estimator ${\Delta d}^{av}$ computed from ${\Delta \varphi }^{av}$ which combines all the phase difference estimation approaches as ${\Delta \varphi }^{av}={(\Delta \varphi }^{ori}+{\Delta \varphi }^{d}+{\Delta \varphi }^{int})/3$. The error associated with the estimation of ${\Delta d}^{av}$ is also shown in Figure 4. As observed, ${\Delta d}^{av}$ provides a more accurate prediction of the displacement than either approach alone.

**Figure 5:**
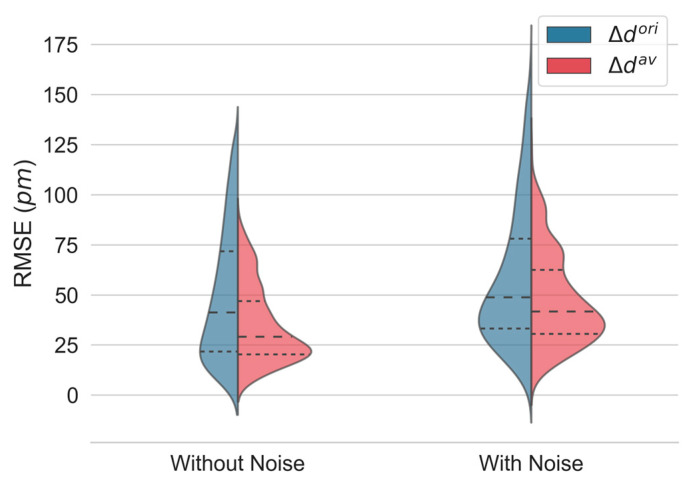
Kernel density estimates of the root-mean-square error (RMSE) of displacement estimation in simulated interferograms without and with noise using the original (${\Delta d}^{ori}$) and the proposed estimator (${\Delta d}^{av}$). The median (dashed) and the first and third quartiles (dotted) are shown.

The RMSE was used to evaluate the quality of each estimator. The kernel density distributions of the RMSE of the displacement computation for all depth locations, obtained with ${\Delta d}^{ori}$ and ${\Delta d}^{av}$ in simulations without and with noise, are shown in Figure 5. The proposed approach performs better, with a pronounced decrease in the median error and interquartile range (IQR). Detailed values are given in Table 1.

As expected, a decrease in performance was observed for both methods when noise was added to the generated interferogram. Nevertheless, the proposed estimator (${\Delta d}^{av}$) performs significantly better than the original one (${\Delta d}^{ori}$). 

### 4.2. Minimum Detectable Displacement

**Figure 6:**
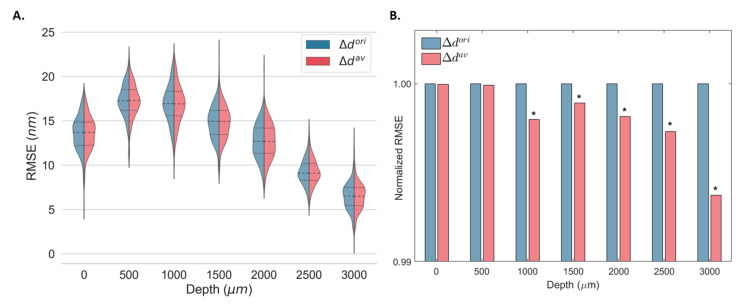
Kernel density estimates of the root-mean-square error (RMSE) displacement distribution for 256 depth locations (**A**) and mean normalized RMSE (**B**) of a static gold mirror at multiple optical path differences (OPD) using the original (${\Delta d}^{ori}$) and the proposed estimator (${\Delta d}^{av}$). The median (dashed) and the first and third quartiles (dotted) are shown. * Statistically significant at *p* < 0.001.

Both ${\Delta d}^{ori}$ and ${\Delta d}^{av}$ were used to estimate the displacement from the same elastography data in static conditions (real data) at different OPDs and their corresponding minimum detectable displacements. The kernel density distributions of the RMSE associated with the displacement estimation at 256 different lateral locations using ${\Delta d}^{ori}$ and ${\Delta d}^{av}$ are shown in Figure 6A. Figure 6B shows the normalized RMSE of all locations for all OPDs. As demonstrated by the numerical simulation, the error associated with displacement estimation was equivalent for ${\Delta d}^{ori}$ and ${\Delta d}^{av}$ for small displacements, and both converged to zero when no displacement was induced (Figure 4). Thus, for static measurements, equivalent error distributions were expected for both methods (Figure 6A). Nevertheless, a significant decrease in the overall RMSE was obtained for OPDs larger than 1000 μm (lower SNR).

The minimum detected displacement at different OPDs was obtained from $\sigma_{{\Delta \varphi }^{ori}}$ and $\sigma_{{\Delta \varphi }^{av}}$ at 256 different locations using Equation (1) (Table 2). For OPDs equal to and greater than 1000 μm, an improvement in the accuracy of the displacement estimation was achieved using ${\Delta d}^{av}$ estimation. Improvements between 0.01% and 0.37% were observed depending on the imaging depth. No significant changes were observed for optimal SNR (OPD = 0 μm).

### 4.3. Phantom Displacement

**Figure 7:**
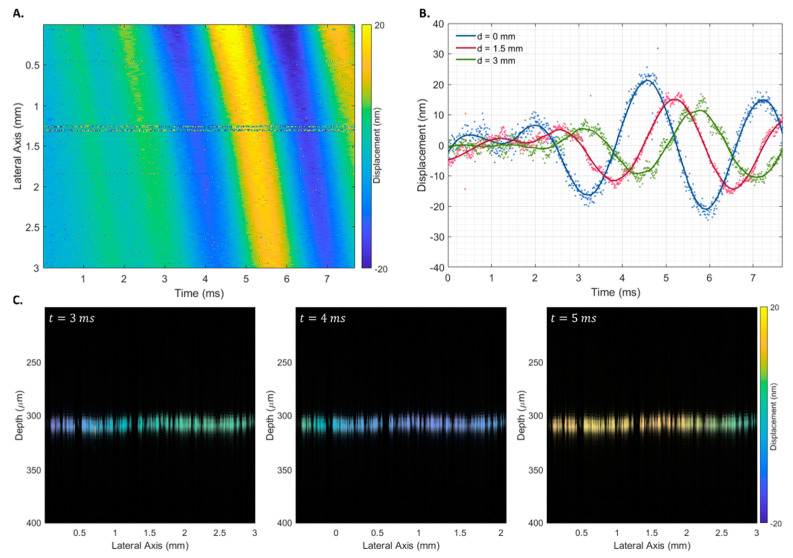
Spatiotemporal displacement map of a 10 g/L homogeneous agarose phantom after a 500 μs transient pulse (**A**), displacement curves at three lateral locations at progressively higher distances from the piezoelectric actuator (**B**), and Rayleigh wave propagation at times 3, 4, and 5 ms (**C**). The phase difference was estimated using the proposed estimator (${\Delta d}^{av}$).

The proposed displacement estimator (${\Delta d}^{av}$) was used to infer the elastic properties of agarose phantoms with different elastic properties. Figure 7 shows the spatiotemporal displacement map for a phantom with an agarose concentration of 10 g/L after a transient pulse of 500 μs (Figure 7A), displacement curves at three lateral locations at progressively higher distances from the piezoelectric actuator (0, 1.5, and 3 mm) (Figure 7B), and the structural B-scan of the phantom’s interface superimposed with ${\Delta d}^{av}$ at three times (3, 4, and 5 ms).

**Figure 8:**
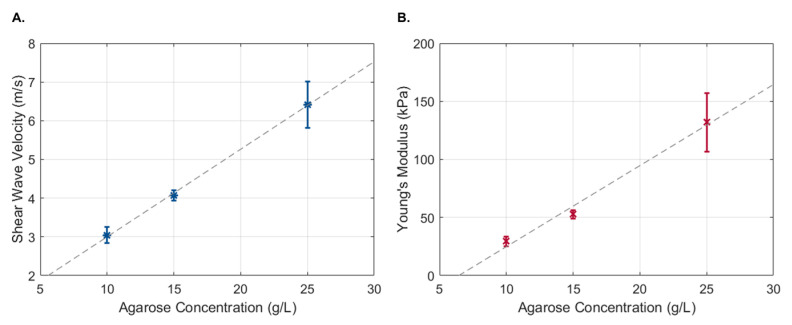
Shear wave propagation velocity (**A**) and Young’s modulus (**B**) as a function of agarose concentration. Data are presented as the mean ± standard deviation.

The propagation of the Rayleigh wave velocity was calculated using the displacement estimation based on the ratio between the propagation distance and the time to reach the maximum displacement. No discernible differences were observed when using transient pulses of different widths. Therefore, all 18 measurements were used to obtain the shear wave propagation velocity of each phantom. As expected, a linear increase in shear wave velocity was observed with an increase in the phantom’s agarose concentration (Figure 8A), which corresponded to an increase in the phantom’s Young’s modulus (Figure 8B).

## 5. Discussion

Quantitative evaluation of tissue elastic properties by phase-resolved OCE depends on the measurement noise, the precision of the phase difference estimation and hence the tissue displacement, and the method used to derive the tissue’s Young’s modulus. Measurement noise is intrinsically related to the phase stability of the system. This can be improved by modifying the instrument setup, typically by introducing timing references. In this study, an MZI clock and FBG-triggering were used to improve timing synchronization and achieve temporal stabilities consistent with those reported in the literature [11,13,25]. Various approaches have also been proposed to reduce noise sensitivity and improve the accuracy of phase difference estimation. However, these rely heavily on depth (often combined with lateral) averaging of the interferogram data, which reduces their resolution.

In this study, we characterize the performance of phase difference estimation as a function of the magnitude of the tissue displacement and the depth location of the scatterer. The accuracy of the original phase difference estimation method (${\Delta \varphi }^{ori}$) was evaluated, as well as that of phase-difference estimators derived from: (i) the first-order derivative (${\Delta \varphi }^{d}$), (ii) the integral (${\Delta \varphi }^{int}$) of the interferogram data, and (iii) the combination of three phase-difference estimators (${\Delta d}^{av}$). These estimators were tested on simulated OCE data in which the displacements could be precisely adjusted and were known *a priori*. We observed that the initial depth location of the scatterer affected the precision of the ${\Delta d}^{ori}$, ${\Delta d}^{d}$, and ${\Delta d}^{int}$ estimators. Each approach was the most accurate in 1/3 of the initial depth locations tested. Since the initial location of the scatterer is not known from real measurements, we predicted that the best displacement estimation would be achieved using a combination of all approaches. 

We demonstrated that ${\Delta d}^{av}$ was the most accurate displacement estimator in the simulated data. For small displacements, both methods achieved similar accuracy. However, as the simulated displacement increased, the error associated with the estimation was lower for ${\Delta d}^{av}$ than for ${\Delta d}^{ori}$. This was possible due to the complementary and opposite trends of the ${\Delta d}^{ori}$ estimation and those from phase-invariant mathematical variations of the original interferogram (${\Delta d}^{d}$ and ${\Delta d}^{int}$). While the latter overestimated the simulated displacement with increasing displacement, the former underestimated it. Overall, a reduction in the median RMSE associated with displacement prediction of approximately 70% and 85% was observed for simulated data without and with noise, respectively. Despite the relatively modest gain, our goal is to use OCE to detect changes in the mechanical properties of the retina that may be associated with Alzheimer’s disease and other neurodegenerative disorders before structural changes can be detected. Regardless of its magnitude, any correction will bring us closer to our goal.

No significant changes were expected for static OCE data, as both methods converge to zero in the absence of displacement. However, ${\Delta d}^{av}$ resulted in an improvement in the displacement estimation depending on the initial relative depth location of the sample and, consequently, the SNR. The ${\Delta d}^{av}$ resulted in a lower RMSE than ${\Delta d}^{ori}$ for imaging depths at OPDs greater than 1000 μm, where the SNR is lower. Comparatively lower minimum detectable displacements could be measured in signals with low SNR. Thus, we have shown that the proposed method can significantly improve the resolution of displacement estimation from phase data. It is noteworthy that the typical correlation between the measured phase difference and the OCT SNR ($\sigma_{\Delta \varphi }\approx 1/\sqrt{SNR}$) [6] was not found. However, since we aim to compare the accuracy of both methods, the absolute values of the minimum detectable displacements do not have a direct impact.

The feasibility of the proposed method to estimate tissue displacement under dynamic conditions was also evaluated. Based on ${\Delta d}^{av}$, the shear wave velocity was estimated for phantoms with different elastic properties. As expected, an increase in agarose concentration resulted in an increase in the shear wave velocity and Young’s modulus. Although the benefits of increased displacement measurement accuracy in wave-based OCE may not be readily apparent, they can have a significant impact on elasticity estimation when considering numerical methods. An example is the application of Newton’s method to a wave solution represented by the method of fundamental solutions [26]. We have recently shown that, when using Newton’s-type iterations to recover the elastic properties of the medium, 1% noise in the data can lead to a two- to three-orders-of-magnitude higher error in the recovered elastic coefficients [26].

We have illustrated that we can develop novel solutions to improve the accuracy of phase estimation by considering external factors such as the magnitude of the tissue displacement and the depth location of the scatterer. We have also illustrated that the proposed estimator, obtained by combining three estimators for the phase difference, although modest in gain, improves the accuracy of displacement estimation without the loss of temporal or axial resolution. The main drawback of the proposed method is the increase in computational time. However, it is important to mention that the improvement comes at no cost since no changes to the optical setup or the instrument are required. The proposed method is also easy to implement, independent of the system setup, and can even be applied to previously recorded data. 

## Figures and Tables

**Table 1:** Detailed values of the displacement root-mean-square error (RMSE) distribution in simulated interferograms without and with noise using the original (${\Delta d}^{ori}$) and the proposed estimator (${\Delta d}^{av}$). Data with noise are presented as the mean ± standard deviation. * Statistically significant at *p* < 0.001.

		1st Quartile	2nd Quartile	3rd Quartile	IQR
**Without Noise**	${\Delta d}^{ori}$(*pm*)	23.1	42.3	70.5	47.5
	${\Delta d}^{av}$(*pm*)	20.6	29.9	48.1	27.6
	%	89.3	70.7	68.3	58.1
**With** **Noise**	${\Delta d}^{ori}$(*pm*)	38.9 ± 6.1	57.2 ± 7.4	83.7 ± 7.0	44.8 ± 6.7
	${\Delta d}^{av}$(*pm*)	33.8 ± 6.5 *	49.1 ± 8.2 *	68.1 ± 9.4 *	34.3 ± 5.8 *
	%	86.8 ± 7.1	85.5 ± 5.2	81.1 ± 4.7	76.3 ± 9.5

**Table 2:** Minimum detectable displacements at different optical path differences (OPDs) computed using the original (${\Delta d}^{ori}$) and proposed (${\Delta d}^{av}$) estimators. Data are presented as the mean ± standard deviation of 256 lateral locations at each OPD.

		Minimum Detectable Displacement (nm)		
		${\Delta d}^{ori}$	${\Delta d}^{av}$	%
**OPD**	0 μm	13.58 ± 1.99	13.58 ± 1.98	100.00 ± 0.05
	500 μm	17.26 ± 1.88	17.25 ± 1.88	99.99 ± 0.08
	1000 μm	16.86 ± 2.18	16.85 ± 2.18	99.91 ± 0.44
	1500 μm	14.78 ± 1.98	14.77 ± 1.98	99.95 ± 0.33
	2000 μm	12.67 ± 2.01	12.65 ± 2.02	99.89 ± 0.59
	2500 μm	9.21 ± 1.44	9.19 ± 1.44	99.87 ± 0.16
	3000 μm	6.46 ± 1.61	6.43 ± 1.61	99.63 ± 0.72

## Data Availability

The code and raw data supporting the conclusions of this manuscript will be made available by the authors upon formal and reasonable request.
